# Evaluation of Renal Function in Obese Children and Adolescents Using Serum Cystatin C Levels, Estimated Glomerular Filtration Rate Formulae and Proteinuria: Which is most Useful?

**DOI:** 10.4274/jcrpe.galenos.2018.2018.0046

**Published:** 2019-02-20

**Authors:** Dilşah Önerli Salman, Zeynep Şıklar, Eda Nisa Çullas İlarslan, Z. Birsin Özçakar, Pınar Kocaay, Merih Berberoğlu

**Affiliations:** 1 Ankara University Faculty of Medicine, Department of Pediatrics, Ankara, Turkey; 2 Ankara University Faculty of Medicine, Department of Pediatric Endocrinology, Ankara, Turkey; 3 Ankara University Faculty of Medicine, Department of Pediatric Nephrology, Ankara, Turkey

**Keywords:** Obesity, glomerular filtration rate, body cell mass, cystatin C

## Abstract

**Objective:**

There is a growing interest in the relationship between obesity and renal damage. The effect of obesity on renal function in children and adolescents has not been adequately investigated. In addition, there is no complete consensus on the reliability of various renal function parameters. The primary goal of this study was to evaluate renal function in obese children and adolescents using glomerular filtration rate (GFR), cystatin C, and creatinine (Cr)-derived formulas. We also compared classical GFR measurement methods with methods based on bioimpedance analysis-derived body cell mass (BCM).

**Methods:**

We enrolled 108 obese and 46 healthy subjects aged 6-18 years. Serum cystatin C, serum Cr, 24-hour proteinuria, Cr clearance, and GFR were evaluated in both groups. Estimated GFR was measured with Cr-based, cystatin C-based, combined (cystatin C and Cr) and BCM-based formulae. Both actual and fat-free mass body surface areas (BSA) were used when required. Metabolic parameters (blood glucose, insulin, and lipids) were analyzed in the obese subjects. International Diabetes Federation criteria were used to identify metabolic syndrome (MetS).

**Results:**

We did not detect statistically significant differences between the obese and control groups for mean Cr (p=0.658) and mean cystatin C (p=0.126). Mean cystatin C levels of MetS patients were significantly higher than those of non-MetS obese participants (p<0.001). Cr-based GFR measurements, BCM-based measurements and a combined Cr and cystatin C measurement showed a statistically significant increase in the GFR of obese subjects compared to controls (p=0.002 and p<0.001). This increase was negatively correlated with duration of obesity. Estimations based on actual or fat-free mass BSA did not differ either. Only the Filler equation showed a statistically significant decrease in eGFR in MetS patients. There were no statistically significant differences between the obese and control groups for proteinuria (p=0.994) and fat-free mass proteinuria (p=0.476).

**Conclusion:**

We conclude that cystatin C could be used as an earlier biomarker than Cr in the detection of impaired renal function in obese children, especially those with MetS. Cr-based formulae reveal hyperfiltration as the first change in renal function. Decreasing eGFR seen in MetS patients with cystatin C-based formulae, but not Cr-based formulae, may represent the early stages of renal damage. Using fat-free mass or BCM for eGFR formulae in obese children seems to provide no additional information.

**What is already known on this topic?**

The effects of obesity and metabolic syndrome on kidney function in the child and adolescent age groups have not been adequately examined. There is insufficient data concerning the degree of impairment of renal function and its clinical significance. There is also no consensus on the parameters that assess renal function most reliably.

**What this study adds?**

Cystatin C could be used as a biomarker which detects impaired renal function at an earlier stage than creatinine (Cr) in obese children, especially those with metabolic syndrome (MetS). Cr based formulae detect hyperfiltration as the first change in renal function. Decreasing estimated glomerular filtration rate (GFR), seen with cystatin C-based formulae, in MetS patients, may represent the early stages of renal damage. Using fat free mass or body cell mass for estimated GFR formulae in obese children appears to provide no additional information.

## Introduction

Obesity is a serious health problem that adversely affects whole-body systems, particularly the cardiovascular and endocrine systems (1). Among the adverse effects of obesity, kidney problems have recently begun to attract more attention. Increased obesity-related glomerulopathy has become apparent in the last 20 years as the role of obesity in the onset and progression of adult kidney disease has been better understood (2). In the last 30 years, with increased obesity prevalence, a significant increased prevalence of chronic kidney disease (CKD) and end-stage renal failure has been observed (2). Vivante et al (3) found that overweight and obesity were serious risk factors for end-stage renal failure in their 30-year survey of 1.2 million adolescents. Obesity was also found to be associated with negative effects on the allograft and reduced allograft survival in patients undergoing renal transplantation (4). 

Despite this growing interest the effects of obesity and metabolic syndrome (MetS) in children and adolescents on renal function have not been sufficiently investigated. In addition, there is no consensus on the reliability of renal function parameters and which of these best represents “true” renal function (4). Glomerular filtration rate (GFR) is one of the most important parameters used to determine renal function and can be calculated with different formulae. GFR is generally calculated using body surface area (BSA)-based formulae. However, these calculations may give incorrect results, particularly for obese children, due to a higher BSA than in normal-weight children. Cystatin C is a biomarker recommended for use in GFR calculations because it is easily glomerular-filtered, has a low molecular weight and is not dependent on muscular mass (5,6). Studies have indicated that cystatin C-derived formulae provide more accurate results than conventional GFR calculation methods (6). However, both conventional GFR formulae and cystatin C-derived formulae may be affected by the amount of adipose tissue, so that calculation of GFR based on non-adipose tissue is considered a more accurate method (7). Proteinuria, one of the best predictors of renal damage, is another parameter that should be considered in renal function evaluation (8).

The primary goal of the present study was to extensively evaluate renal function in obese children and adolescents, using serum cystatin C concentration, cystatin C- and creatinine (Cr)-based eGFR and measures of proteinuria. We also investigated the relationship between these parameters and MetS components and obesity duration. Furthermore, we compared classical GFR measurement methods with those based on bioimpedance analysis-derived body cell mass (BCM) and fat-free mass BSA.

## Methods

### Study Design

This prospective, observational study was conducted between January 2014 and January 2015 at the Pediatric Endocrinology Outpatient Clinic of Ankara University Faculty of Medicine. All participants or parents gave informed consent prior to participation. Institutional Ethics Committee Approval was obtained (Ankara University Ethics Committee, decision dated: 23 September 2013, no: 14-540-15, for the study entitled “Control of renal function in obese children and adolescents and relation with MetS components”). Project support was obtained from the Association of Pediatric Endocrinology and Diabetes.

### Patient Enrollment

We enrolled consecutive patients aged 6-18 years with a body mass index (BMI) >95^th^ percentile. We excluded patients with comorbidities (diabetes mellitus, congenital heart disease and chronic systemic disorders) and those who were receiving systemic drugs at the time of presentation. Normal-weight (BMI <85^th^ percentile) healthy subjects constituted the control group.

### Measurements and Outcomes

The demographic data (age, gender and duration of obesity) and physiological measurements [weight, height, height standard deviation score (SDS), BMI, blood pressure, and pubertal stage] of the participants were recorded. Laboratory evaluations were performed, including fasting plasma glucose, blood Cr, blood total cholesterol, blood low-density lipoprotein cholesterol (LDL-C), blood high-density lipoprotein cholesterol (HDL-C), blood triglycerides, and 24-hour urine protein and urine Cr levels by using automated Roche^®^ Moduler (Germany). Fasting plasma insulin measured by radioimmunoassay. Cystatin-C was measured by nephelometric immunoassayby using BNII^®^ Nephelometer (Siemens, Germany). The homeostatic model assessment-insulin resistance (HOMA-IR) of each patient was calculated. HOMA-IR levels of >2.22 in prepubertal girls, >2.67 in prepubertal boys, >3.82 in pubertal girls and >5.22 in pubertal boys were accepted as demonstrating insulin resistance (9). The body-fat mass of each participant was measured with a bioimpedance analyzer (Tanita^®^ BC 418) to compare GFR and cystatin C levels with BCM and Cr clearance (CrCl). Both BSA (for CrCl) and fat-free mass BSA (adopted using total fat-free mass in GFR formulae as body weight) were analyzed. BCM was calculated as intracellular fluid divided by 0.70 (7). We identified MetS patients, 10-18 years old, based on the International Diabetes Federation (IDF) MetS criteria (10). The IDF criteria define MetS as central obesity (waist circumference >90^th^ percentile) combined with any two of the following: dyslipidemia (triglycerides >150 mg/dL), reduced HDL-C (<40 mg/dL), increased blood pressure (systolic >130 mmHg or diastolic >85 mmHg), increased fasting plasma glucose (>100 mg/dL), or previously diagnosed type 2 diabetes.

For the GFR measurements, we used four groups of formulae: Cr-based, cystatin C-based, combined (Cr- and cystatin C-based), and BCM-based (Table 1) (11,12,13,14,15,16,17,18). We used only Cr-based formulas for GFR measurements with fat-free cell mass.

For evaluation of proteinuria, 24-hour urine samples were collected. Protein excretion of 100 mg/m^2^/day indicated a nephritic status, while >1 g/m^2^/day indicated a nephrotic status (19). Fat-free mass adjusted proteinuria was also calculated.

### Statistical Analysis

Statistical analysis was performed using SPSS software (SPSS version 20.0 for Windows; SPSS Inc., Chicago, IL, USA). Continuous variables were expressed as mean ± SD or median (minimum-maximum), and nominal variables were expressed as numbers (%) in the descriptive analyses. Percentage comparisons of groups were performed using the chi-square test and multivariate logistic regression analyses were performed for statistically significant variables. Sperman’s rho correlation was used. Normality of data was tested using the Kolmogorov-Smirnov test. Normally distributed variables were compared using the t test, and non-normally distributed variables were compared using the Mann-Whitney U test. For all statistical analyses, p<0.05 was considered significant.

## Results

### Clinical Characteristics of Participants

A total of 154 children and adolescents were enrolled in the study. Of these, 108 consituted the obese group and 46 made up the control group. The age and gender distributions of the two groups were similar although there was a higher proportion of subjects in puberty compared with the control group (see Table 2). Unsurprisingly weight (p<0.001), height (p<0.001), BMI (p<0.001), BMI SDS (p<0.001), height SDS (p<0.001) and waist circumference (p<0.001) were greater in the obese group than in the control group (Table 2). Laboratory analysis of all cases are given in Table 3. Based on the IDF criteria, MetS was identified in 14.8% of the obese participants and in none of the control participants.

### Creatinine and Cystatin C Results

Serum Cr and cystatin C levels were compared to evaluate renal function. There were no statistically significant differences in mean levels of Cr and cystatin C between the all obese (p=0.658) and control groups (p=0.126) (Table 4). The mean concentration of cystatin C in the obese children with MetS was significantly higher than in the controls and the non-MetS obese participants (p<0.01). 

We performed Spearman’s correlation and a regression analysis to evaluate the factors affecting cystatin C and Cr levels. There was a positive correlation between cystatin C and total cholesterol (r=0.275, p=0.001), LDL-C (r=0.277, p<0.001), triglycerides (r=0.318, p<0.001) and fasting insulin (r=0.255, p=0.001). There was an inverse correlation with HDL-C (r=−0.219, p=0.006). There was no significant correlation between Cr and total cholesterol (r=–0.085, p=0.296), LDL-C (r=–0.098, p=0.225), HDL-C (r=0.091, p=0.260), triglycerides (r=0.11, p=0.889), fasting plasma glucose (r=0.016, p=0.840), and fasting insulin (r=0.133, p=0.101).

### Renal Function Evaluation Based on Glomerular Filtration Rate Formulae

GFR results in the obese patients were calculated with CrCl, fat-free mass CrCl, Bedside Schwartz et al (13), Andersen (7), Donadio et al (18), and Donadio et al (17) formulae. The results were significantly higher in the obese group than in the control group. In the obese group without MetS, the GFR results calculated with the CrCl and Bedside Schwartz formulas were significantly higher than those in the control group (p<0.05). The GFR values calculated with the cystatin C-derived Filler formula and the cystatin C and serum Cr-derived Bouvet formula were lower in the MetS-diagnosed obese patients than in the non-MetS obese patients and the controls (p<0.05). In both the MetS obese group and the non-MetS obese group, GFR levels calculated with fat-free mass CrCl, and the formulae of Andersen (7), Donadio et al’s (18), and Donadio et al’s (17) formula were higher than those of the control group (see Table 5). Correlation of the duration obesity and the changes of GFR were analysed. As the duration of obesity increased, GFR calculated with both the Donadio et al’s (17,18) formulae were increased, but GFRs calculated with Filler (p=0.008), Bouvet (p=0.020), and Bedside Schwartz (p=0.038) showed a decrease.

### Renal Function Evaluation Based on Proteinuria

There were no statistically significant differences between the obese and control groups for proteinuria (p=0.994) and fat-free mass proteinuria (p=0.476) (Table 6). There were also no statistically significant differences between the MetS obese, non-MetS obese and control groups with regard to proteinuria and fat-free mass proteinuria results. Nephritic-range proteinuria was detected in 12 non-MetS obese participants (11.1%) and in six control-group participants (12%). Nephrotic-range proteinuria was not detected in any of the participants.

## Discussion

Obesity has a deleterious effect on renal function, so the capability to determine exact renal function is more important in obese patients than in those of normal weight. One of the most useful parameters of renal function is eGFR. Accurate calculation of GFR has a vital role in the accurate identification of kidney disease, drug-dose calculations, CKD management and prognosis (20). There are several models for GFR measurements, but none is accepted as the gold standard for GFR calculation (7). There is a potential risk that Cr-based formulae may yield GFR results that are even lower in obese patients than in normal-weight individuals. Since CrCl is subject to variability due to a number of causes including acute and chronic disease, it is reported that this method is not very sensitive for GFR (21). However, up to 80% of clinical laboratories use CrCl as the main method for determining GFR (20). It is accepted that serum cystatin C gives more accurate GFR results because it is less affected by muscle mass and diet than Cr based methods (22). Roos et al (23) compared 24 cystatin C and Cr studies involving a total of 2,007 participants. They found that at a 95% confidence interval and according to the Moses-Littenberg linear regression model, cystatin C was more interoceptive for indicating renal dysfunction compared to Cr [cystatin C: 3.99 (3.41-4.57) versus Cr: 2.79 (2.12-3.4)] (23).

There is an increasing number of studies investigating eGFR and Cistatin-C measurements in children. Miliku et al (24) compared the relationship between body composition and eGFR calculated from both Cr and cystatin-C concentrations. They found that, eGFR was influenced by BMI and BSA. Moreover, eGFR calculated on the basis of Cr concentrations, was also influenced by lean mass percentage and fat mass percentage of the patients. This study was limited to six year-old healthy children. In another study, Correia-Costa et al (25) evaluated 163 normal and 150 overweight/obese children, between eight and nine years of age. They compared eGFR, CrCl, Cr and cystatin-C levels of the patients. Results showed that, overweight/obese children had lower eGFR values using several formulae except when using CrCl and the Schwartz formula.

In the present study, kidney function of obese participants was assessed with Cr-based, cystatin C-based, combined Cr and cystatin C and BCM-based GFR formulae and with proteinuria levels. We calculated the BCM and fat-free mass of obese participants from BSA-based GFR measurement techniques based on the hypothesis that the increased BSA of these participants may lead to inaccurate results. In a new model, Andersen (7) found that both the BCM and the weight models are reliable methods for estimating GFR in children, with a higher accuracy than the currently recommended Schwartz model. To the best of our knowledge, our study is the first to use the Andersen method. We did not find any differences between using the BCM model and CrCl methods. However, we obtained similar results using fat-free cell mass for GFR calculations with Cr-based formulae. We obtained higher GFR values in the obese group compared to the control group using calculations with combined Cr and cystatin C (Donadio et al. (17)) and all BCM or Cr-based formulae. We consider that the increased GFR with Cr-based formulae found in this study support the hyperfiltration and renal-function effects in obese participants. However, there was no difference between GFR rates using the cystatin C-based formula of Filler and Lepage (14) or Zappitelli et al (15) We believe that this is due to similar cystatin C levels between the obese and control groups.

We detected higher cystatin C levels in the MetS obese group compared to the non-MetS obese group, as an indicator of renal damage. Cystatin C is recommended as an interoceptive biomarker indicating kidney function when Cr levels are not yet affected, such as during the early stages of kidney damage and with mildly decreased GFR (7). Cystatin C is less affected by muscle mass and diet compared to Cr and so should be used instead of Cr for more accurate GFR measurements (22). Research by Marwyne et al (26) showed that cystatin C gave more accurate results compared to Cr in abnormal GFR measurements when compared to 99mTc-diethylenetriamine pentaacetic acid (r=0.526, p=0.001).

Some pediatric researchers have made comparisons between cystatin C and Cr when predicting renal damage. In five of 12 studies done using receiver operating characteristic analyses, it was confirmed that cystatin C was significantly more sensitive than Cr, but another five studies did not find any statistically significant difference between the biomarkers. In the remaining two studies, statistical comparisons were not performed. One study reported cystatin C to be significantly better than Cr while Cr was not superior to cystatin C in any of these 12 studies (7). Our results showed that elevated serum cystatin C is an earlier biomarker than elevated serum Cr in the detection of impaired renal function in obese children. Furthermore, in cystatin C-based formulae, a steady decline in GFR parallel to the duration of obesity may be noted, which may be an indication that functional damage was superceeded by structural damage over time. Based on these results, we conclude that Cr-based formulae may not reflect real renal function, because of a tendency to give inaccurate higher GFRs, particularly during the early stages of renal damage in obese patients. 

With regard to GFR estimations using Cr- or cystatin C-based formulae, the question of whether decreased GFR in obese children may be overlooked with increased cystatin C concentrations, when using these formulae, arises. We believe that since cystatin C concentration increases with renal function impairment, it can be useful when GFR begins to decrease. 

Dyslipidemia is a metabolic parameter that indicates increased risk of renal failure. As a result of reabsorption of fatty acids and cholesterol from tubular epithelial cells, tubulointerstitial inflammation may stimulate foam cell formation and tissue damage. At the same time, dyslipidemia may damage mesangial cells and glomerular capillary endothelial cells, such as podocytes. Both hypercholesterolemia and hypertriglyceridemia may lead to podocyte damage. Accumulation of lipoproteins in the glomerular mesangium may stimulate matrix production and glomerulosclerosis (8). This hypothesis led to the idea of investigating the effect of dyslipidemia in the etiology of CKD. In a study by Servais et al (27) on 925 dyslipidemic patients, cystatin C values were significantly higher in patients with MetS than in patients without (0.86±0.23 vs 0.79±0.20 mg/L, p 0.0001) and were correlated with dyslipidemia (p<0.001). In our study, in accordance with the literature, cystatin C values were found to be significantly higher in patients with MetS; the Spearman analysis showed positive correlation between cystatin C and total cholesterol, triglycerides and LDL-C, but a negative correlation with HDL-C. This result suggests that cystatin C is more accurate than Cr as a biomarker for detecting the negative effects of dyslipidemia on renal function in obese children.

When we assessed our results in terms of proteinuria, we found no statistically significant difference between the obese and control groups. In addition, we found no statistically significant difference between the control and obese groups with and without MetS. Proteinuria and microalbuminuria are accepted as indicators, as well as risk factors, for chronic renal failure (28). BMI is the second most common factor after proteinuria in increased risk of end-stage renal failure. Obesity-related renal disease involves a wide spectrum of disorders, from excretion of urinary albumin to proteinuria and/or decreased GFR. The adverse effects of fat accumulation on kidney hemodynamics and obesity-related glomerulopathy are two important possible mechanisms for this. Hemodynamic changes cause inflammation, oxidative stress, apoptosis and finally, the development of renal scarring (29). The absence of significant differences between our study groups in terms of proteinuria indicates that no apparent structural renal damage had begun in our participants during the study period. Studies examining the relationship between renal protein loss and MetS have reported that increased albuminuria and proteinuria or the presence of microalbuminuria are risk factors for MetS (8). However, the accepted conclusion in the current literature is that protein loss does not increase the risk of MetS, unlike chronic renal failure development (30). We believe that proteinuria is not useful for indicating renal function impairment in obese pediatric and adolescent patients.

### Study Limitations

Measurement of inulin clearence, which is a valuable tool for GFR estimation, was not performed in our study groups.

## Conclusion

Serum cystatin C can be used as an earlier biomarker than Cr-based GFR estimations in the detection of impaired renal function in obese children, especially those with MetS. In a comparison of GFR measurement formulae, we found that Cr-based formulae may give normal or higher GFR results, particularly during the early stages of renal dysfunction in obese children. In addition cystatin C may be a more sensitive biomarker, when compared to Cr-based GFR estimations, for detecting dyslipidemia-mediated renal impairment in obese children. Proteinuria is not an appropriate early biomarker for indicating damaged renal function. It also appears that there is no need to use fat-free mass or BCM for determining eGFR in obese children.

## Figures and Tables

**Table 1:**
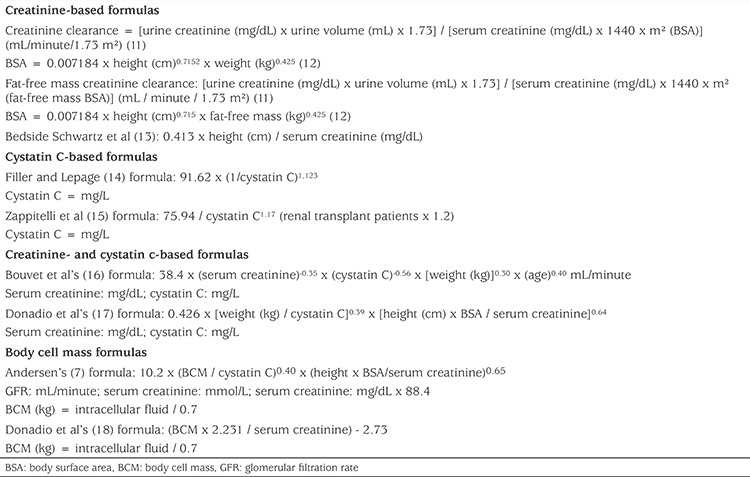
Formulae used for glomerular filtration rate calculations (7,11,12,13,14,15,16,17,18)

**Table 2:**
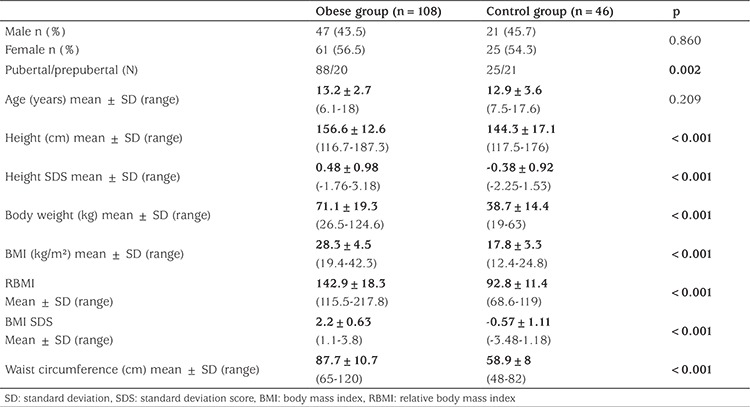
Clinical characteristics of all subjects and controls

**Table 3:**
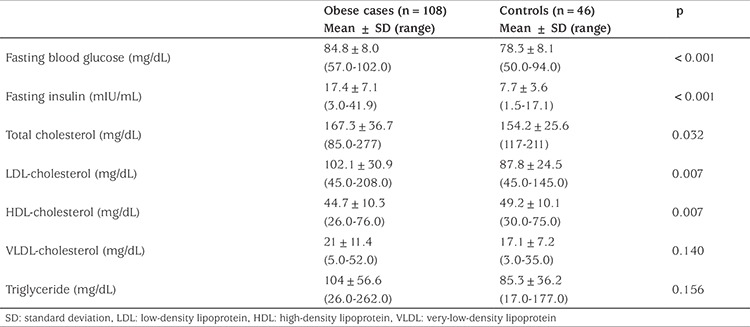
Laboratory characteristics of all cases

**Table 4:**
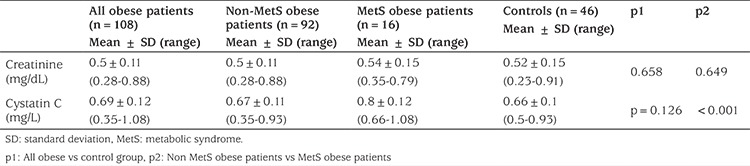
Serum creatinine and cystatin C levels

**Table 5:**
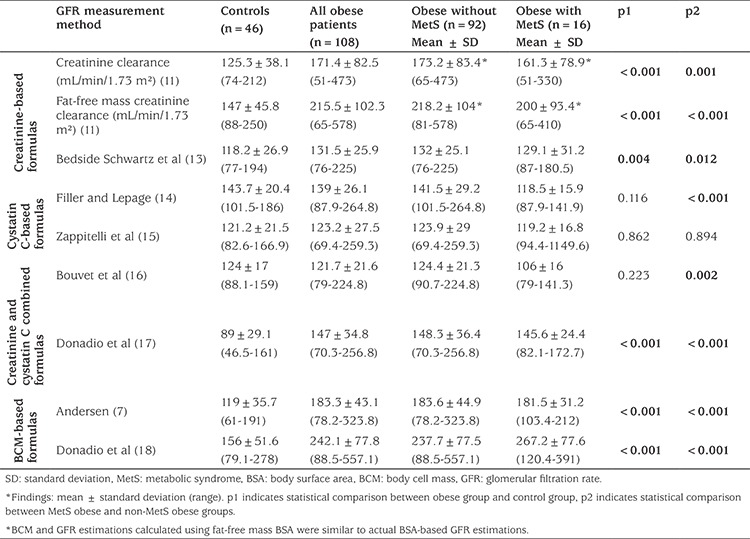
Comparison of glomerular filtration rate measurement methods in obese subjects with and without metabolic syndrome and the control group

**Table 6:**
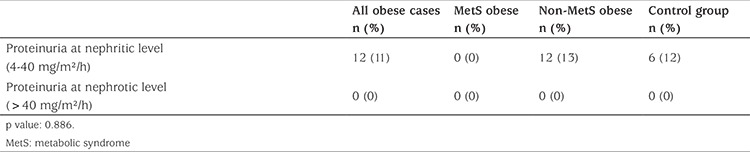
Proteinuria in obese cases with or without metabolic syndrome
